# Determination the Levels of Subjective and Observer Rating of Drowsiness and Their Associations with Facial Dynamic Changes

**Published:** 2017-01

**Authors:** Mohsen POURSADEGHIYAN, Adel MAZLOUMI, Gebraeil NASL SARAJI, Ali NIKNEZHAD, Arash AKBARZADEH, Mohammad Hossein EBRAHIMI

**Affiliations:** 1. Research Center in Emergency and Disaster Health, University of Social Welfare and Rehabilitation, Tehran, Iran; 2. Dept. of Occupational Health, School of Public Health, International Campus, Tehran University of Medical Sciences, Tehran, Iran; 3. Dept. of Mechatronics, Faculty of Engineering, Islamic Azad University South Tehran Branch, Tehran, Iran; 4. Dept. of Biostatistics, School of Medicine, Tehran University of Medical Sciences, Tehran, Iran; 5. Occupational and Environmental Health Research Center, Shahroud University of Medical Sciences, Shahroud, Iran

**Keywords:** Driver drowsiness, Facial dynamic changes, ORD, KSS

## Abstract

**Background:**

We determined the levels of subjective and observer drowsiness and facial dynamics changes.

**Methods:**

This experimental study was done in the virtual reality laboratory of Khaje-Nasir Toosi University of Technology in 2015. Facial dynamics changes like changes in eyes, mouth and eyebrows were surveyed on twenty-five drivers in 2015byKSS (Karolinska Sleepiness Scale) and ORD (Observer Rating of Drowsiness). ANOVA Repeated Measure and MANOVA Repeated Measure tests were used for data analysis. Also, neural network and Viola-Jones were used to detect facial characteristics. PERCLOS (Percentage of Eye Closure), blink frequency and blink duration were inspected for eyes parameters. The size of open mouth during drowsiness was inspected for mouth parameter. During the inspection of eyebrow, the number 50 denoted eyebrow in normal position. For eyebrows above the normal position, a range of 50 to 55 was specified; in addition, 45–50 was found as the specified range for eyebrows under normal position.

**Results:**

Descriptive statistics of the dynamic changes in mouth and eyes illustrated that during the driving process, the level of sleepiness increased as well as changes of eyes and mouth. However, statistical findings during car driving revealed that dynamic changes in eyebrows had clear expression with a constant trend. Similar studies on data obtained from KSS and ORD showed that both of these parameters simultaneously increased as well as the level of drowsiness. In addition, a significant relationship existed between facial expression and drowsiness.

**Conclusion:**

This research would be an effective and efficient tool for timely alarming and detecting the drowsiness quickly and precisely.

## Introduction

Detecting drowsiness can aid the reduction of a number of fatal road accidents. Studies have shown that more than 1.3 million and 20 to 50 million people are killed and injured in road accidents, respectively (1). Statistics issued by the US National Highway Traffic Safe Administration (NHTSA) have indicated that 100000 car crashes happen every year which is only caused by driver sleepiness. Road accidents cost over 12.5 billion which resulted in 1550 mortalities and 71000 injuries (2).

Car accidents in urban and sub-urban areas are mainly caused by human factors. According to statistics, human error is the main reason for a range of 90 to 95%of car accidents. It is assumed that drivers fatigue causes 25% of accidents and particularly 60% of fatal or injurious road accidents (3). In a research conducted by National Highway Traffic Safety Administration (NHTSA) with 107 randomly selected vehicles, the driver drowsiness was the principal reason of 58% of car accidents (4).

In Britain the drivers fatigue is the main cause of20% of the road accidents (5). In recent years, smart systems and the use of wireless sensors have been broadly utilized for monitoring and transferring the vehicle and driver’s situation (WSN). Smart vehicles have improved the quality of driving. This improvement is the result of computer program and software designs for gear, brake system, steering wheel etc. AD hoc networks were initial systems that developed automatic steerage in vehicles delay in responding to the changes in ambient space was the weakness of these systems (6, 7). When the driver is driving the vehicle, time is an important factor for appropriate response. Numerous techniques have been developed by famous car manufacturing companies to detect the level of driver’s drowsiness particularly during night time. Several weaknesses and advantages are attributed to these techniques. Techniques developed for announcing driver fatigue are categorized in three sets:

1. Mathematical and statistical surveillance systems,
2. Vehicle monitoring systems,
3. Crew by using sensor networks and
4. Smart systems for driver’s fatigue detection.

In 2009, a system was developed by Hosking and Liu to detect the level of alertness (8).This system detects driver’s fatigue using a facial features-based model. Also, signals as the threshold of sleep were extracted based on EEG waves analysis in Barbato et al study (9).Connor et al studied the risk of accidents of large vehicles like tracks caused by drivers’ drowsiness and suggested techniques based on intelligent systems in 2002 (10). Maislinand Dinges introduced intelligent control steering system when the driver is fatigued which is consistent with new the technology (11). The process of monitoring and immediate surveillance was done by Jo et al to forecast driver’s fatigue (12). Bergasa created the immediate surveillance system and smart monitoring in 2006 (13). The blinking rate, as a new method for monitoring and controlling driver’s drowsiness, was suggested by Johns in 2003 (14). He then tested the level of drowsiness by shining infra-red rays and analyzing the reflected rays in 2007(15).

Driver’s drowsiness detection techniques have been used in several investigations in order to reduce the road accidents; such systems detect the sleepiness and alertness of the driver by shacking the steering ball or the seat (16).The following parameters were used to detect drowsiness:

1. Based on physiological signals (ECG and EEG) (17)
2. Based on of driver performance (number of line crossing and car spacing) (18)
3. Based on facial expression (19).

Physiological methods detect high precision drowsiness, perfectly. However, in these methods some sensors should be set on the body which might be annoying for the user. In methods based on driver performance so much time is required and consequently micro-sleeps are not detected. Researchers mostly used inspection of apparent changes to detect drowsiness (20).

The behaviors of drivers are obviously seen in apparent changes of their head and facial expressions.

Longer blinking, slow movements of eyelids, staring, yawning and frequent head dropping are usual characteristics of drowsy driver considered by researchers in sleepiness detection (18, 21, 22). In addition, in some methods, the assessment of the driver subjective drowsiness was done based on KSS (Karolinska Sleepiness Scale) criterion and calculating the level of interpretive drowsiness was done by the ORD(Observer Rating of Drowsiness) (23,24). According to the discussions, complex systems can detect drowsiness; since in these systems different methods are simultaneously utilized to reduce the weaknesses of the methods. As far as there was no complex and plenary system, the purpose of this research was to employ simultaneously methods on the basis of the changes of eyes, mouth and eyebrows and find the relationship between these parameters with KSS and ORD so as to promote the precision and accuracy of the data collected in the drowsiness detection

## Methods

This experimental research was carried out among Twenty-five professional suburban drivers in virtual reality lab of Khaje-Nasir Toosi University of Technology in 2015.Inclusion criteria were having no eyesight weakness (wearing no glasses), two years of driving experience and normal appearance (with no abnormal beard and mustache). In this regard, eligible subjects were selected randomly.

**Fig. 1:**
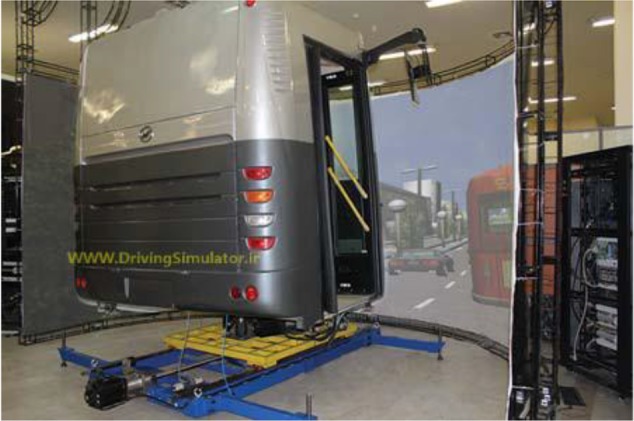
Driving simulator model AKIA-BI 301

Virtual-reality driving simulator in a temperature and audial controlled quiet room was provided to take photos of the driver. This study was done on driving simulator model AKIA-BI 301 (Fig 1)(25).When drivers began to drive the simulator, some facial photos were taken to find out drowsiness. Before the test, they were asked to drive a vehicle tentatively for some minutes. Then, subjects were requested not to take care of unrealities of the simulator, and drive based on traffic rules.

A camera was mounted at the start point, to take photos from subjects’ facial features and continuously, KSS appeared on the road every ten minutes. ORD was done by the observer every ten minutes at the same time. The test was conducted between9 A.M to 12 A.M in order to control for circadian rhythm (26).Controlled light of vehicles ahead was simulated by bright light shock, which led to the reduction of subjective sleepiness. In order to prevent interruptions during the test, camera and simulator were continuously monitored. Mounting the camera and simulator continuously, prevented the interruptions. After all wheels exited the road, the test ended. Then, a software was built for receiving images of eyes, eyebrows and mouth as well as for checking the dynamic changes of facial features according to the information. Dynamic changes in facial features has been based on eye closure (PERCLOS), eye blink duration and eye blink frequency. By recording deviations from normal situations, eyebrows and mouth were being tracked. After matching and synchronizing, available data from the previous studies were used in rudimentary model from facial dynamic changes.

Viola- Jones algorithm, as a popular quick algorithm, was used to detect objects immediately and facial expressions (particularly the area of eyes) as well as to facilitate the detection of drowsiness. Head, in this algorithm, is detected as an area with oval shape by the ratio of its diameters with the hole of eyes and the final target is the option with similar color and hue of human (27).

Considering the position of eyes in the face, the upper part of the right eye along with the left eye were surveyed. Then, the changes in the white part and pupil colors were studied. In order to detect open and close eyes and blinking as well as reducing the data volume, the images of the eyes were changed into binary form. In an open eye, the ratio of dark pixels of the upper part to the lower part is greater than in a closed eye, which is due to dark colors of pupil and eyelashes. A normalization based on illumination was done before converting the image into binary form. Also, erosion and dilation operators were employed for removing tiny black spots. Finally, the ratio of black pixels related to the whole pixels of upper and lower parts was calculated and used to detect close eyes.

Changes of size of open mouth due to yawning, was the main reason for approximating the open month in the present study. The place of mouth was transferred to Fuzzy C-Means (FCM) unit which is a clustering method in which a part of data is subdivision of two or more clusters. In this phase, calculations pertained to centers of clusters and membership functions in spectral amplitude. A calculation method of the correct number of clusters was required for independent functioning. This target was achieved by using numerous iterations of FCM for a spectrum of hypothesis numbers of clusters along with selection of eligible parts on the basis of validity of cluster. In the next step, this information was transferred to special FCM. In order to remove noise from the image, the special information produced by FCM was used. For specification of a cluster to one-pixel, defuzzification was done after convergence of c-FCM and results revealed an output of FCM in a binary format image that led to mouth detection.

For inspecting the accuracy of mouth detection process, also, two more tests were conducted. The first test was begun from central part of lips and the second test, calculated value of angle between the position of lips and area between the eyes. The central area of lips is perpendicular to the area between eyes.

The ratio of area of mouth and degree of open mouth was employed for detecting the size of open mouth in different frames as the formula:

(1) DoO=w/h=w/(h×cosΘ)

Where

“w”= breadth of the rectangle (the distance between two corners of lip)
“h”= the height of the rectangle of the lips calculated as the distance between upper and lower lines

Changes in the rectangle open mouth coupled with yawning can be derived through calculations. As expected, the size of open mouth specifically changed while yawning (28).

During the inspection of eyebrows, the number 50 denoted eyebrow in normal position. A range of 50 to 55 was specified for eyebrow above the normal position and a range of 45 to 50 was for eyebrow below the normal position. So, highest eyebrow is +5 and lowest eyebrow is −5. The output of this section is transferred to another software section for extraction and recognizing the characteristics of detected areas in previous section. These traits were considered as driver’s characteristics and were saved in personal files provided by the software. Inspecting recorded frames were done for surveying the level of dynamic changes of facial features. Finally, data were merged based on the level of drowsiness, KSS and ORD. Due to multivariate analysis of these variables during the time, the requirements for detecting drowsiness and level of sleepiness were provided via a statistical model.

## Results

The method was applied on 32 suburban bus drivers aged in a range of 26 to 60 years old that 25 of them got drowsy during the test. As the driving began, photos were taken by a camera placed in front of the driver and ORD was done every ten minutes. Also, data from KSS was simultaneously obtained every ten minutes. Then, the relation between the information of different parameters was investigated. Demographic characteristics of these drivers presented in Table 1.

**Table 1:** Demographic Characteristics of participants

**Demographic Characteristics**	**Mean±SD**	**Minimum**	**Maximum**
Age	37.2±10.95	26	60
BMI	24.03±2.64	19.6	29.4
Work experience	10.32±7.39	2	25
Years of smoking	7.26±6.4	0	20

### Eyes Features during Drowsiness

**Fig. 2:**
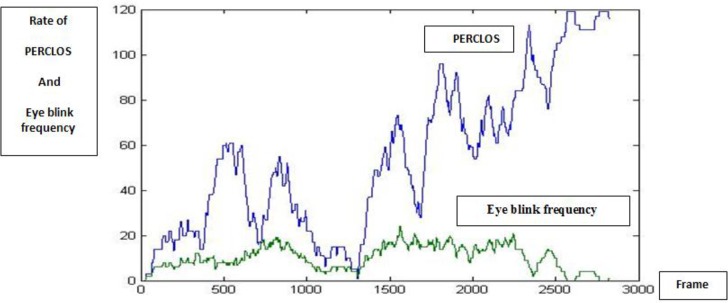
Blinking rate and PERCLOS in a part of frame

According to the signals of eyes, MLP neural system was used for detection and determination of level of drowsiness. There were two in-puts in the neural system: first, in order to get perfect features of eyes and information of drowsiness, eyes were closed. Secondly, the rate and duration of blinking in times and information of drowsiness were perfect. As shown in Fig. 2, PERCLOS and blinking rate had upward and downward trends, respectively, when the driver was tired as time progressed (Fig. 2).

**Fig. 3:**
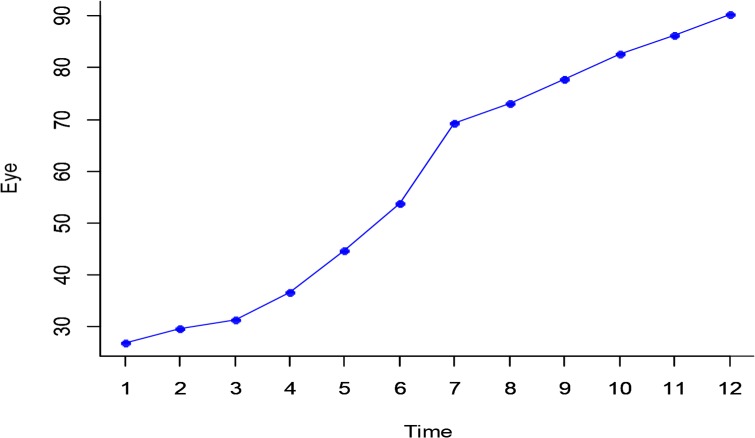
changes of eyes dynamics in time

Having inspected the descriptive statistics of dynamics of eyes, a considerable increase in eyes drowsiness with increased time was observed. From the rejecting sphericality assumption, Greenhouse-Geisser test was used to validate changes. A considerable change was observed at significance level of 5% of eyes dynamics in time, (F_2.74, 65.86_=135.26, *P*<0.001, Partial Eta^2^=0.849).In other words, it was claimed that a significant effect existed on the time and the level of drowsiness had increased in time. Also, the Eta squared coefficient showed that about 85% of total variance of this model was determined by this variable.

### Mouth Features in Drowsiness

**Fig. 4:**
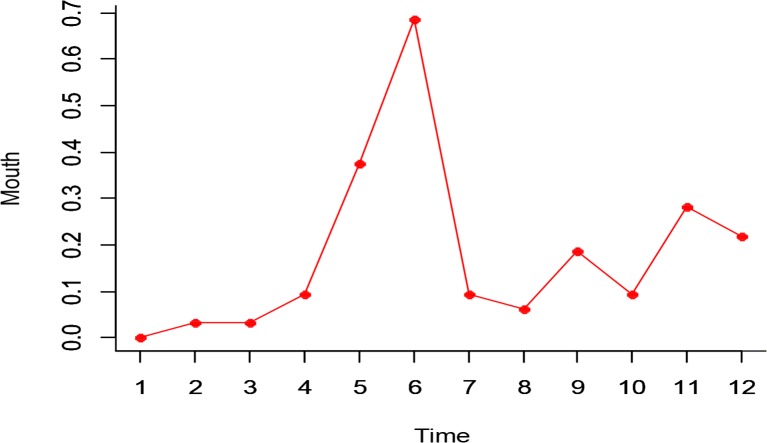
Dynamic changes of mouth in time

According to Figure 4, a significant relationship was indicated between mouth dynamics and progression of time. In addition, based on Greenhouse-Geisser test, there was a significant change in mouth dynamics in time (F_3.47,8.47_=89.59, df=2.42, *P*<0.001, Partial Eta^2^=0.789).

### Eyebrow Dynamics during the Drowsiness

**Fig. 5:**
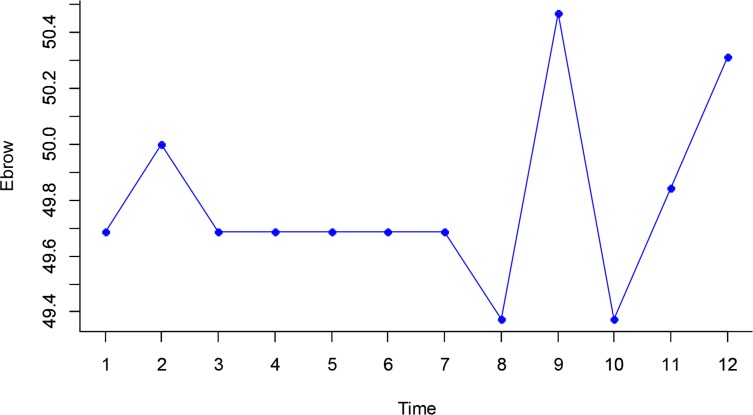
Dynamic changes of eyebrows in time

Fig. 5 shows the dynamic changes of eyebrows in time. No significant relationship was observed between time progression and dynamic changes of eyebrows, according to the statistical analysis.

### Data from KSS and ORD during the Drowsiness

**Fig. 6:**
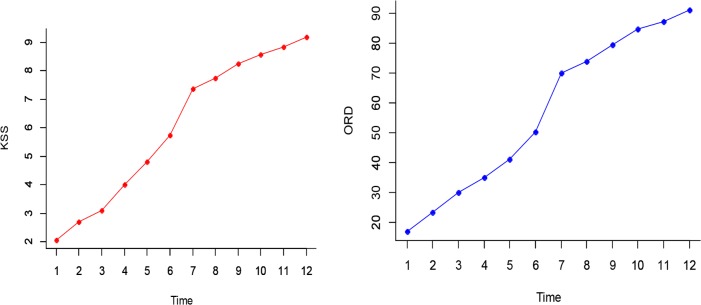
Changes of ORD and KSS in time

Both KSS and ORD had direct relationship with the level of drowsiness and after 60 minutes an abrupt increase occurred (Fig.6). To survey the relationship between levels of sleepiness, KSS, ORD, MANOVA Repeated Measure were employed. Results indicated that time had significant effect on both KSS and ORD. It is noteworthy these two variables explained 98% of the total variance. The linear combination of both dependent variables over the research periods were significantly different (F_(22,10)_=22.52, *P*<0.001; Wilks’lambda= 0.02; Partial Eta^2^=0.98). Based on KSS calculations, There was a significant difference between changes of drowsiness in time (F_(3.8,90.4)_ =178.3, *P*=0.001, Partial Eta^2^=0.81), as well as for ORD (F_(3.8,90.4)_ =178.3, *P* = 0.001, Partial Eta^2^=0.79). Pairwise comparisons by bonferroni correction were done due to the importance of time. The results of both KSS and ORD indicated that many of pairs were significantly different.

### The Relationship between the Results of KSS and ORD and Dynamics of Eyes, Mouth and Eyebrows

MANOVA Repeated Measure, KSS and ORD were used so as to find if any relationship between dynamic changes of eyes, mouth and eyebrows during driving, and changes in the level of drowsiness. Findings showed that time variable had a significant impression on the dynamic variables of facial expressions (eyes and mouth) as well as KSS. Over 99% of the changes of variance allocated to these variables. In this regard, there were significant relationships between dynamic changes of facial features and ORD; also, between linear combination of both dependent variables in 12 periods of the research (Table 2).

**Table 2:** Data related to KSS, ORD and their relationship with dynamics of eyes, mouth and eyebrow

**Factors**	**Wilks’lambda**	**F_(22,3)_**	***P***
Eye – ORD	0.0004	367.62	< 0.001
Mouth – ORD	0.0002	513.98	< 0.001
Eyebrow – ORD	0.001	234.3	< 0.001
Eye – KSS	0.001	175.72	< 0.001
Mouth – KSS	0.001	250.1	< 0.001
Eyebrow – KSS	0.002	73.25	< 0.001

## Discussion

Driver’s drowsiness causes changes in the eyes and facial features and many techniques and algorithms have been developed in order to recognize facial features that detect sleepiness (19). Lopar and Ribarichave shown how quick and accurate Violla-Jones algorithm as a tool was in tracking facial features (20). In this research, image processing based on Violla-Jones algorithm was employed to recognize facial features, detect driver fatigue and determine level of driver alertness. Also, KSS, ORD and parameters of dynamic changes of eyes, mouth and eyebrow were utilized in order to improve the accuracy of recognitions. Belz et al’s study by employing KSS and ORD found a significant relationship between the levels of drowsiness (29). Results of multiple variable tests indicated the impressive effect of time on KSS and ORD. Both of the mentioned variables provided 98% of the changes in variance.

Eyes dynamic changes can be considered as a key parameter for fatigue detection and numerous inspections have been done on it. Dynamic changes of eyes had a direct relationship with the level of sleepiness, according to gradient of graphs (30). Kumar and Barwin employed a new method by the use of eyes dynamics, blinking rate and Viola-Jones simultaneously (31).This method could detect 92% of driver’s drowsiness and specified the driver. A direct relationship was observed between the level of drowsiness and eyes dynamics. A level of 5% error in eyes dynamic implied that there was a significant change in time. GROUP INTERACTION test was utilized to provide an appropriate model; linear model provided 89% of the changes of variance. However, dual comparisons test showed that majority of dual differences at the level of 5% with BONFRONI correction were significant.

Ingre et al conducted an inspection on the relationship between eyes dynamic changes and KSS test. They used the information obtained in five-min intervals and concluded that a direct relation had existed between both components (32).In other study, a correlation was found between ORD and dynamic changes of eyes (33) which was in line with our results. Therefore, the accuracy of the results of dynamic changes of eyes in fatigue detection was confirmed.

Mouth dynamic changes are also a target for drowsiness detection inspections. A similar study (34) detected the lips and the size of open mouth for sleepiness recognition using s-FCM clustering. Determination of frequency of yawning could be another way to identify driver drowsiness (28, 35). Therefore, significant correlation was observed between dynamic changes of mouth and time progression. Descriptive statistics of the dynamic changes of mouth in time showed that after abrupt increase in 60th min, there was an increase in yawning frequency rather than previous periods.

KSS and ORD results revealed that a significant correlation exists between the trend of drowsiness level and the dynamic changes of mouth during the test. Unlike the parameters of eyes and mouth, obtained information indicated that dynamic changes of eyebrows had a constant trend in the test. Forehead wrinkles were paid attention as signals of drowsiness in other studies (36). Because, while driver feels sleepy, she/he puts eyebrows above the normal position and keeps her/his eyes open. This change was considered as a forecast result of their study. At a 95% confidence interval, the results of Greenhouse-Geisser test showed no significant difference between eyebrows dynamics during driving, against of pilot study (37). But, based on KSS and ORD correlation whit eyebrow, a significant relationship was observed between dynamic changes of eyebrow and the trend of drowsiness, this result as the same of pilot study (37).

The target of this study was to build a hybrid facial drowsiness detecting model. This research had power points and weaknesses. One advantage in the use of this technique was that it recognized intelligent sleepiness by analyzing various criteria, long time intervals and drive’s background to detect sleepiness. It helps to detect driver drowsiness in real time and to give essential announcements and decrease car accidents. Precision of this method relied on enough illumination and appropriate place of camera. This method also processed the real time with high accuracy in comparison with other methods. Another weak point was that we could not be sure about driver’s sleep, because some of drivers were going to have fake sleeps and finished their tests earlier than others and they could not change their positions too much and also, detection of driver’s eyes in bright light was not possible.

## Conclusion

KSS and ORD analysis proved the dynamic changes in eyes and mouth parameters while driver was sleepy. This method could be an effective and efficient tool for quickly and precisely drowsiness detections.

## Ethical considerations

The approval of Tehran University of Medical Sciences was obtained for conducting the study. All participants were presented about the objectives of the study, and their informed consent was obtained.
